# Treatment Planning Strategies for Interstitial Ultrasound Ablation of Prostate Cancer

**DOI:** 10.1109/OJEMB.2024.3397965

**Published:** 2024-05-08

**Authors:** Pragya Gupta, Tamas Heffter, Muhammad Zubair, I-Chow Hsu, E. Clif Burdette, Chris J. Diederich

**Affiliations:** Department of Radiation OncologyUniversity of California San Francisco 8785 San Francisco CA 94115 USA; Acoustic MedSystems Savoy IL 61874 USA; Department of Neurology and Neurological SciencesStanford University 6429 Stanford CA 94305 USA

**Keywords:** Bioacoustic-thermal models, interstitial ultrasound, prostate cancer, thermal therapy, treatment planning

## Abstract

Purpose: To develop patient-specific 3D models using Finite-Difference Time-Domain (FDTD) simulations and pre-treatment planning tools for the selective thermal ablation of prostate cancer with interstitial ultrasound. This involves the integration with a FDA 510(k) cleared catheter-based ultrasound interstitial applicators and delivery system. Methods: A 3D generalized “prostate” model was developed to generate temperature and thermal dose profiles for different applicator operating parameters and anticipated perfusion ranges. A priori planning, based upon these pre-calculated lethal thermal dose and iso-temperature clouds, was devised for iterative device selection and positioning. Full 3D patient-specific anatomic modeling of actual placement of single or multiple applicators to conformally ablate target regions can be applied, with optional integrated pilot-point temperature-based feedback control and urethral/rectum cooling. These numerical models were verified against previously reported ex-vivo experimental results obtained in soft tissues. Results: For generic prostate tissue, 360 treatment schemes were simulated based on the number of transducers (1-4), applied power (8-20 W/cm2), heating time (5, 7.5, 10 min), and blood perfusion (0, 2.5, 5 kg/m3/s) using forward treatment modelling. Selectable ablation zones ranged from 0.8-3.0 cm and 0.8-5.3 cm in radial and axial directions, respectively. 3D patient-specific thermal treatment modeling for 12 Cases of T2/T3 prostate disease demonstrate applicability of workflow and technique for focal, quadrant and hemi-gland ablation. A temperature threshold (e.g., Tthres = 52 °C) at the treatment margin, emulating placement of invasive temperature sensing, can be applied for pilot-point feedback control to improve conformality of thermal ablation. Also, binary power control (e.g., Treg = 45 °C) can be applied which will regulate the applied power level to maintain the surrounding temperature to a safe limit or maximum threshold until the set heating time. Conclusions: Prostate-specific simulations of interstitial ultrasound applicators were used to generate a library of thermal-dose distributions to visually optimize and set applicator positioning and directivity during a priori treatment planning pre-procedure. Anatomic 3D forward treatment planning in patient-specific models, along with optional temperature-based feedback control, demonstrated single and multi-applicator implant strategies to effectively ablate focal disease while affording protection of normal tissues.

## I. Introduction

Prostate cancer is the second most common cancer in men, with 299010 new cases and 32250 deaths estimated for 2024 in the United States [1], and approximately 1276000 new cases and 359000 deaths estimated worldwide [2]. The standard of care treatment of localized prostate cancer has often involved therapies such as active surveillance, surgical prostatectomy, and/or external beam radiation therapy, brachytherapy, and androgen deprivation therapy, with the particular regimen dependent upon the severity and classification of the disease [3]. While these approaches have demonstrated efficacy in controlling local disease, they come with significant risks of morbidity, including urinary incontinence and erectile dysfunction [4]. With early diagnosis rates on the rise due to advanced screening methods (e.g., biopsy, MRI, PSA), treatment approaches have evolved to encompass a spectrum of options that balance disease control with minimizing treatment-related side effects. Among these approaches, focal therapy has emerged as a promising strategy that seeks to precisely target local cancerous areas within the prostate gland using forms of radiation therapy, targeted drugs, or thermal ablation, while minimizing collateral damage to surrounding healthy tissue and reducing the potential for adverse effects [5], [6], [7]. Thermal ablation [8], [9], [10], [11] as a means of focal therapy has acquired substantial attention in recent years as clinical evidence continues to demonstrate its efficacy for prostate disease. Focal prostate thermal ablation [12] is primarily indicated for patients with localized low-grade prostate cancer, and can be applied with percutaneous or interstitial approaches such using radiofrequency (RF) [13], laser [14], microwave [15] energy sources or cryoablation [16], as well as endocavity transrectal high-intensity focused ultrasound [17] and MR guided transurethral ultrasound TULSA [18]. The transrectal and transurethral ultrasound ablation systems offer superior spatial control of the energy deposition compared to the interstitial technology, albeit with a more time consuming and complex integration. Clinical studies have shown that localization of thermal ablation to intermediate and moderate stage disease can be a safe and effective alternative to whole gland therapy [19], [20]. Further, salvage thermal ablation of localized radiorecurrent prostate disease can be successfully applied using endorectal HIFU and MRg Transurethral ultrasound with good outcomes and safety profiles [21], [22], [23], [24].

Interstitial and intraluminal ultrasound devices, designed for percutaneous placement or positioning within a body lumen/cavity, respectively, have been investigated for thermal ablation therapy in sites such as brain, liver, gall bladder, pancreas, prostate, spine and [25], [26] Due to the properties of ultrasound as an energy source (small wavelength, transducer technology), these devices can have better dynamic and spatial control of the ablation pattern compared to many of the other modalities and [25], [26]. Catheter-cooled interstitial ultrasound applicators, based on linear arrays of tubular transducers within a plastic brachytherapy implant catheter, affords dynamic control of heating along the applicator length, good therapeutic penetration, and directional or angular control of the energy pattern [27], [28], [29]. In consideration of prostate therapy, interstitial ultrasound has potential advantages – the angular patterns and length of heating can be pre-selected and positioned to avoid directing energy to the rectum and urethra and localize therapeutic temperatures and thermal dose to the target. Earlier implementations were used for hyperthermia therapy delivery in conjunction with HDR brachytherapy for clinical studies including treatment of prostate cancer, which clearly demonstrated enhanced energy penetration and conformality, as well as directing energy as a means to protect the rectum [30], [31], [32]. Interstitial ultrasound applicators for thermal ablation are in the early stages of commercialization, with the TheraVision delivery platform and Acoustix applicators (Acoustic MedSystems, Savoy, IL) currently 510(k) FDA cleared for soft-tissue ablation of sites such as liver and brain, although exclusive of prostate. It is found that prostate cancer is multifocal in a majority of cases and targeting large dominant lesions will be feasible, safe and well tolerated compared to applying treatment to whole gland while minimizing the risk of side effects [33]. In the context of prostate ablation, interstitial ultrasound devices may provide for options to deliver precise and selective focal, quadrant or hemi-gland ablation by using single or multiple applicators to target multifocal disease or a single defined intraprostatic lesion [34].

Treatment planning [35], [36], [37] and computational models are important tools currently under development for many modalities to guide and tailor interstitial thermal ablation procedures and may provide for safer and more effective outcomes [38], [39], [40]. These planning and modeling tools can determine optimal designs and patterns of use, and through patient specific treatment planning optimize the device operating parameters and placement to selectively improve the tumor or target ablative temperature and thermal dose coverage. In consideration of interstitial ultrasound and the work reported herein, comprehensive computational modeling approaches have been investigated to calculate ultrasound energy deposition, bioheat transfer, and thermal damage profiles for thermal ablation with single and multiple catheter-based interstitial ultrasound (CBUS) devices [41], [42]. Further, Prakash et al. [43] explored various models for heat dependent dynamic changes in the tissue thermal and acoustic properties, and its impact on the size of ablation zones in prostate and liver with interstitial and transurethral applicators. Modeling of the angular or directional power deposition and heating patterns has been established [44], [45], [46] including dynamic angular control or mechanical rotation. In related work for intraluminal devices in prostate, Burtnk et al. [47] developed treatment planning strategies specific to MRI-guided transurethral ultrasound therapy and investigated on the size and temperature of the accompanying endorectal cooling device (ECD), ultrasound power and frequency control, and treatment margins to reduce thermal injury and improve treatment coverage. These studies demonstrate the applicability of the required modeling with inherent approximations and tissue property selections, and which can best represent interstitial ultrasound ablation in prostate. The findings from these modeling studies can provide the framework for the required development of a treatment planning platform specific to interstitial ultrasound thermal ablation of prostate.

The objective of the present study is to develop a treatment planning platform specific to the clinical implementation of interstitial ultrasound ablation for treatment of focal prostate cancer using the TheraVision system. Expanding on recent modeling studies, a prostate-specific tissue model which incorporates dynamic changes to acoustic and biothermal properties is developed and applied to generate lookup tables with 3D thermal dose and temperature distributions as a tool for a *priori*-treatment planning to optimize the applicator positioning and parameters. 3D finite-difference time domain (FDTD) acoustic-biothermal models are used to estimate the transient temperature and thermal dose distributions in the computational domain. Patient-specific planning with segmentation of prostate and cancer volumes, along with rectum and urethra from anatomic imaging, is applied to demonstrate performance and clinical utility. Two types of temperature-based feedback control, emulating implementation of monitoring points with invasive sensors or MRTI, along with the urethral catheter cooling, are described and applied in example cases as methods to plan and improve therapeutic profiles.

## II. Methods

### A. Background Description of Interstitial Ultrasound Applicators and TheraVision Delivery Platform

**Fig. 1.**
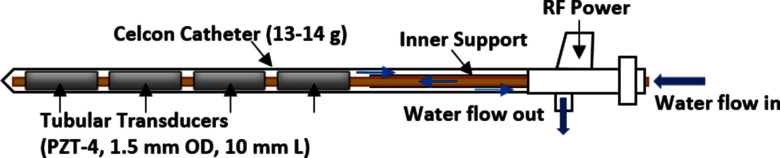
Catheter-based interstitial ultrasound applicator with controlled power deposition in angular direction and length.

Previous studies have shown catheter-based interstitial ultrasound applicators [27], [29], [31] are designed featuring linear arrays of tubular transducers (1.5 mm OD, 10 mm length), inserted within a 13 g plastic implant catheter (2.4 mm OD). These transducers are independently powered and spaced 2 mm apart within a linear array configuration (1-4 elements). The transducers can be sectioned to provide directional or angular control of beam output, typically 90, 180 or for 360 degrees. Further, the transducer assembly and energy direction can be rotated within the catheter as positioned within the tissue. The operational frequency of the ultrasound transducers falls within the range of 6.5–9.0 MHz. A circulating water system is employed to facilitate applicator cooling and efficient coupling, as shown in Fig. 1. Depending on the specific characteristics of the identified target volume, such as size, shape, and location, either 360° or directional (90°, 180°) applicators can be selected. Directional applicators (90°, 180°) provide the control over the direction which can be utilize for directing energy away from the critical organs like urethra, rectum, and the neurovascular bundles (NVB). In addition, independently powered number of elements allow for the control of the ablation along the length of the applicator. The previously developed TheraVision (Acoustic MedSystems, Savoy, IL) is the delivery platform for applying multichannel RF power, applicator water-flow, thermocouple temperature measurement, and image-based geometric treatment planning. The system can import MRI/CT Dicom data of pre-treatment imaging or real-time US imaging. The main functionality of the planning tool includes the 3D volume generation, segmentation of the organs of interest and target regions, and measure approximate dimensions. Based on the geometric evaluation, applicator selection and positioning can be optimized.

### B. Acoustic and Bio-thermal Modeling

3D coupled acoustic and bio-thermal models were applied in both a generalized form and patient specific geometries to determine the acoustic intensity, transient temperature, and thermal dose distributions over a range of operational parameters during interstitial ultrasound heating in either soft tissue or the prostatic computational domain. Acoustic intensity distributions from each applicator inside the tissue medium was calculated using the following equation [27], [48]:

\begin{equation*}
I(x,y,z) = {{I}_0}\frac{{{{r}_0}}}{r}{{e}^{ - 2\alpha \left( {r - {{r}_0}} \right)}} \tag{1}
\end{equation*}

where, *r* (m) is the radial distance and *I_0_* (W/m^2^) is the acoustic intensity at transducer surface at radius *r_0_* (m). *α* (Np/m/MHz) is the acoustic attenuation coefficient of the medium and assumed to be equal to the acoustic absorption as all energy is absorbed locally. The cumulative acoustic output is obtained by summing up the contribution of all transducers along the length of the applicator. The intensity, *I_0_*, applied to the active sector (i.e., 360°, 180°, or 90°) only to incorporate the angular expanse due to the longitudinal sectoring of the transducers.

The transient temperature distribution in the tissue medium was modeled using the Pennes bioheat transfer equation [49]:

\begin{equation*}
{\rho}\,_{t}C_{t}\frac{\partial{T}}{\partial{t}}=k\nabla^2{T}-{\omega}_{b}C_{b}(T-T_{b})+Q \tag{2}
\end{equation*}

where, *k* (W/m/°C), *ρ_t_* (kg/m^3^), *C_t_* (J/kg/°C) and *C_b_* (J/kg/°C) are the thermal conductivity, density, specific heat capacity of the soft tissue and blood, respectively. *ω_b_* (kg/m^3^/s) is the blood perfusion rate and *T* (°C) and *T_b_* (°C) represents the soft tissue and arterial blood temperatures, respectively. *Q* (W/m^3^) is the volumetric power deposition due to the absorption of acoustic intensity (1) in the tissue medium, given by:

\begin{equation*}
Q(x,y,z) = 2\alpha I(x,y,z) \tag{3}
\end{equation*}

Iso-effect thermal dose (*TD*) was estimated based on the transient temperature exposure in the soft tissue using the empirical relationship proposed by Sapareto and Dewey [50]:

\begin{equation*}
TD(t) = \int _0^t{{R}^{\left( {{{T}_{ref}} - T(t^{\prime})} \right)}}\mathrm{d}t, \tag{4}
\end{equation*}

where $R = \left\{ \! {\begin{array}{l} {0.25,T(t) < {{{43}}^ \circ }\mathrm{C}}\\ {0.5,T(t) \geq {{{43}}^ \circ }\mathrm{C}} \end{array}}\right. $ and *T_ref_* = 43 °C.

**Fig. 2.**
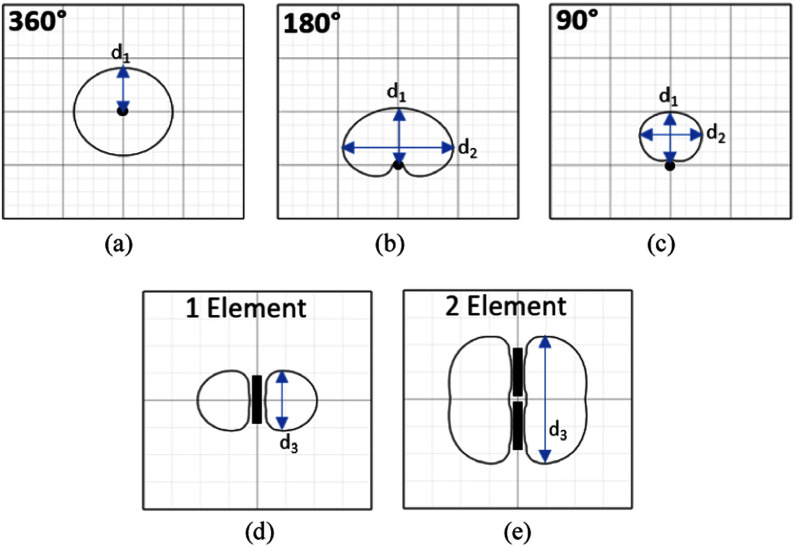
Treatment ablation patterns (*TD*_43_ = 240 min contours) and measurements for applicators 360° (a), directional: 180° (b) and 90° (c), and along the axial direction using one element (d) and two elements (e).

A thermal dose *TD*_43_ ≥ 240 min is considered the threshold for complete tissue necrosis during interstitial ultrasound thermal ablation [51]. Fig. 2 provides an illustrative overview of the treatment ablation patterns and measurements for transverse and axial cross-sections for 360° and sectored directional applicators.

Acoustic and thermal properties used in the present study for different tissues are listed in Table I. Also, the values for *T_b_*= 37 °C and *C_b_* = 3720 J/kg/°C were considered. The properties of the tissue medium were assumed to be homogeneous and constant with temperature change except blood perfusion, which reduced to zero for *TD*_43_ ≥ 300 min [41], [47]. Thermally significant discrete large blood vessels were not modelled. Initial temperature of the tissue was kept as the normal body temperature, *T_0_* = 37 °C and a constant heat flux boundary condition was taken on the edges of the computational domain. A convective boundary condition was assumed at the inner surface of the applicator catheter-wall to model the applicator cooling, given by [43]:

\begin{equation*}
\mathop{n}\limits^{\rightarrow}{}k\nabla{T} = h({{T}_w} - T) \tag{5}
\end{equation*}

where, *T_w_* = 25 °C is the temperature of the cooling water circulated and *h* = 500 W/m^2^/°C is the convective heat transfer coefficient [43].

**TABLE I:** Acoustic and Thermal Properties

Symbol	Properties	Unit	Soft tissue [60]	Prostate [43], [60]	Urethra [60]	Rectum [60]
*α*	Attenuation	Np/m/MHz	5.0	5.3	5.3	5.0
*ρ*	Density	kg/m^3^	1000	1050	1050	1050
*k*	Thermal Conductivity	W/m/°C	0.5	0.54	0.54	0.56
*C_t_*	Specific Heat Capacity	J/kg/°C	3600	3639	3639	3639
*ω_b_*	Blood Perfusion	kg/m^3^/s	2.5	2.5	2.5	3.6

**Fig. 3.**
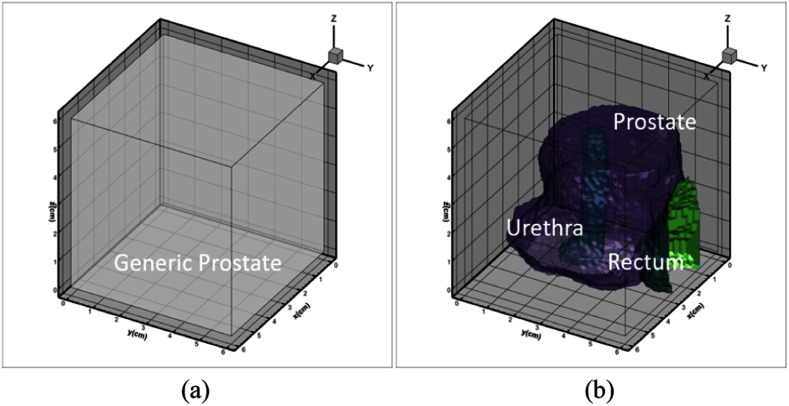
Computational domain for (a) generic prostate model (b) patient-specific tissue model.

Both a generic prostate tissue model and patient-specific numerical models (Fig. 3) were developed [52] and solved the governing acoustic and thermal (1)-(4) along with appropriate boundary conditions using an explicit finite difference scheme in the time domain [53], [54]. Segmentation of the relevant anatomical volumes and target regions were performed manually using MP-MRI/CT Dicom images imported on the TheraVision planning software and then saved as .stl files. These .stl files were voxelized and converted into 3D cartesian domains to define patient-specific tissue volumes and assign the properties accordingly for the acoustic-thermal modelling. Simulations were performed in the 3D cartesian coordinate system where the dimension of the computational domain was taken: 6 cm × 6 cm × 6 cm. A grid independence (convergence test) was performed to optimize and select the grid size of Δ*x* = Δ*y* = Δ*z* = 0.5 mm and Δ*t* = 0.1 s in space and time respectively to provide accurate solutions [53], [54]. The time step stability was determined to be within the Courant–Friedrichs–Lewy (CFL) stability criteria. The computational code was written in C++ programming language and executed on a computer with specifications: RAM 32.0 GB, 64-bit operating system, x64-based processor, Intel(R) Core(TM) i7-6700 CPU @ 3.40 GHz. The numerical solutions of the models were not optimized for computational efficiency, with 1–2 hours of run time required for generic cases, and 1.5–3 hours required for full forward cases with multiple applicators.

### C. Model Validation With Experimental Data

The numerical solver and model were validated against experimental data previously acquired and reported in FDA 510(k) K150019 documentation [55] and within Prakash et al. [41] describing interstitial ultrasound ablation within ex-vivo tissues. The 510(k) experimental data was measured using 360° and directional 180° applicators (9 MHz, 2x10 mm transducers) in a single insertion configuration within muscle. For Prakash et al. [41], data was measured in bovine liver tissue using either two or three 360° applicators (7 MHz, 2x10 mm transducers,) placed 2 cm apart in a parallel or cluster insertion configuration. Briefly for each set of experiments, blocks of tissue were warmed to 37 °C in a saline water bath to mimic human normothermic temperatures and thermal protein denaturation, implanted with the ultrasound applicator(s) for the specific configuration, and sonicated for the test parameters. After each test, the tissue sample was sectioned across the heating plane, and the dimensions of the visible thermal coagulation were measured. A total of 3-9 ablations in separate samples were performed for each test case, and coagulation dimensions with standard deviation reported. The generic computational model developed herein, with appropriate tissue properties and a perfusion of zero, was used to simulate each test configuration within ex-vivo tissue. Acoustic intensities between 7.1–13.5 W/cm^2^ and heating times between 3.0–10.0 mins were applied, following the same as used in the experiments [41], [55]. The calculated *TD*_43_ = 240 and 600 min contours were used to delineate the dimensions of the visible coagulation zone for comparison.

### D. Generic Model – Fast A Priori Treatment Planning

The 3D generalized prostate model was implemented to generate temperature and thermal dose profiles over perfusion ranges and various applicator operating parameters as anticipated for prostate ablation of focal or localized disease. The model parameters are given in Table I, using the values of “prostate” throughout the computational domain. A total of 360 cases were simulated to effectively bracket the user space with variation of applicator directionality (360°, 180°, 90°), the number of active array transducer elements (1–4), acoustic intensity (8–20 W/cm^2^), heating or sonication time (5, 7.5, 10 mins), and tissue blood perfusion (0, 2.5, 5 kg/m^3^/s). Performance metrics (as defined in Fig. 2) for each case were determined and tabulated from the calculated lethal thermal dose distribution, with dimensions of the ablation zone defined by *d_1_* the radial extent, *d_2_* the cross-section width, and *d_3_* the length using the *TD*_43_ 600 and 240 min dose contours. Further, the simulated 3D thermal dose and temperature contours were also integrated as 3D objects for overlay and manipulation of positioning on planning volumes as an a priori planning tool. The calculated 3D data sets of temperature and thermal dose were converted into .nrrd files, where NRRD is a library and file format [56], and used for visualization and image processing within TheraVision. The Visualization Toolkit (VTK) [57] (opensource) within TheraVision was used for displaying data sets of thermal dose and temperature, and manipulating the positioning to best account for safety margin and target coverage.

### E. 3D Patient-specific Forward Treatment Planning

A 3D patient specific modeling module was developed to facilitate more detailed planning for complex cases, or after the implant and positioning of applicator(s) is completed. The prostate, rectal wall, urethra, and target region(s) can be segmented from real-time (US) or pre-treatment images (MRI, US) and assigned properties of Table I. The target region and prostate are centered within a 6 cm × 6 cm × 6 cm model grid. Following selection of applicator parameters (number of transducer segments, power, sonication time), the power deposition pattern is calculated, and a transient solution to the BHTE is used to determine the temperature distributions at the end of the sonication and accumulated thermal dose distributions including a 2 min cool down. A representative series of 12 clinical patient cases with T2 or T3 focal disease are used as examples, and plans were generated based on pre-procedure MP-MRIs with delineation of the target(s). Cases range from localized DILs targeted with a single applicator, to regions of more diffuse or multifocal disease requiring multiple applicator implants. Images were acquired, de-identified and anonymized, and used according to the UCSF IRB Protocol 18-26558 titled “In Silico Simulation Studies with Anonymized Patient Images”. Additionally, urethral cooling and integrated temperature-based feedback control were applied to the modeling as an option, if desired for clinical implementation. Urethral cooling was modeled by applying the condition referred in (5) to the surface of the urethra contour, with a water temperature of 25 °C, representing an irrigated cooling catheter in place. Two types of temperature feedback control were implemented, a binary controller for continuous power modulation or a pilot point controller to stop treatment, and both assume invasive temperature probe placement [58] or MR temperature monitoring [59]. The binary controller regulates applied power for the set treatment duration following *P_on_* for *T* ≤ *T_reg_*, and *P_off_* for *T* > *T_reg_* over 1s update times. The pilot point controller regulates power as *P_on_* for *T* < *T_reg_*, with *P_off_* when *T* ≥ *T_reg_* and treatment is ended.

## III. Results

### A. Model Validation With Experiments

As mentioned in the Section II-C, simulations using the modeling platform were performed and compared against experimental results obtained in ex-vivo tissue studies performed in previously reported investigations. A comparison of simulated to experimental data reported in muscle [55] using 360° applicators and directional 180° applicators in single insertion configuration are shown in Table II (Case 1, 2). Comparison of simulations to experimental data [41] with two and three 360° applicators in a cluster configuration, 2 cm separation, are reported in Table II (Case 3, 4). In each case, experiments were repeated for 3–9 times, with standard deviations ranging between 0.1–0.5 cm. The calculated transverse and axial extents were in good agreement with measurements for single applicators, showing 1.2–2.4 cm transverse extent and 2.3–2.4 cm axial extent within 8% with a reported 0.1–0.2 cm difference. Similarly, there was good agreement for multiple clustered applicators, within ∼10% of 4.5–4.8 cm transverse extent (3.3–4.7 cm in short axis) and 2.7–3.0 cm axial extent with a reported 0.2–0.5 cm difference.

**TABLE II:** Experimental and Simulation Measured Ablation Zone

Case	*Ex-vivo* Tissue	Applicator Setting				Lesion Dimension	
		Conf.-*f*^a^	Intensity (W/cm^2^)	Heating time (min)		Transverse extent (cm)	Axial extent (cm)
1	Chicken Muscle	360-9	7.16	5.0	Experiment [55]	2.4±0.1	2.2±0.1
					Simulation	2.4	2.3
2	Chicken Muscle	180-9	13.3	3.0	Experiment [55]	1.3±0.2	2.2±0.1
					Simulation	1.2	2.4
3	Bovine Liver	360-7 360-7	7.5	10.0	Experiment [41]	4.0 ± 0.3 3.2 ±0.4^b^	2.9 ±0.2
					Simulation	4.5 3.3^b^	2.7
4	Bovine Liver	360-7 360-7 360-7	7.5	10.0	Experiment [41]	4.6 ± 0.5 4.2 ±0.5^b^	3.3 ±0.5
					Simulation	4.8 4.7^b^	3.0

a. Conf.-*f* : Configuration-frequency, ^b^short axis

### B. Fast A Priori Treatment Planning

#### 1) Generation of Planning Data and Lookup Table

The generic prostate-specific tissue model was applied over the practical range of controlling parameters of the interstitial ultrasound applicators for prostate thermal ablation for the 360 test cases, with dimensions of the corresponding thermal coagulation zone (Fig. 2) given in Table S1 in Supplemental Data for the complete data set, and Table III representing a small subset for a two-transducer applicator (*f* = 7.5 MHz, *I_0_* = 12 W/cm^2^) at various power levels and directivity given for context. Over the range of parameters evaluated (1-4 transducers, 8-20 W/cm^2^ acoustic intensity, 5–10 min sonication, 0–5 kg/m^3^s perfusion), desired ablation zones could be preselected ranging from 0.7–3.0 cm (radial penetration) and 0.8–5.3 cm (axial extent), and for directional 180° and 90° applicators the width could range from 0.9–5.4 cm and 0.4–3.3 cm, respectively (Table S1).

**TABLE III:** Partial Lookup Table for Selection of Two-Element Applicators, Listing Dimensions and Volumes of the Thermal Coagulation Zone (*TD*_43_ 240-600 min) for Applicator Directivity, Acoustic POWER, and Time Over the Range of Blood perfusion

Applicator	Acoustic Power (W)	Heating time (min)	Lesion Dim. (cm)^a^ *TD*_43_ = 600 min			Radial Dist. (cm)^a^ *TD*_43_ = 240 min	Vol. of ablation (cm^3^) ^a^ *TD*_43_ = 240 min
			*d_1_* (Rad.)	*d_2_* (Width)	*d_3_* (Ax. L)	*R*	*V_abl_*
360	9	5	1.59 (1.80-1.43)	-	2.68 (2.83-2.57)	1.65 (1.88-1.48)	18.38 (24.55-14.55)
		7.5	1.86 (2.13-1.67)	-	2.95 (3.17-2.79)	1.94 (2.22-1.72)	26.89 (37.78-20.65)
		10	2.05 (2.38-1.84)	-	3.17 (3.48-2.97)	2.11 (2.48-1.89)	34.45 (50.61-25.85)
180	4.5	5	1.52 (1.74-1.35)	2.44 (2.84-2.09)	2.47 (2.58-2.38)	1.59 (1.83-1.39)	6.96 (9.79-5.18)
		7.5	1.78 (2.08-1.57)	2.90 (3.47-2.50)	2.65 (2.84-2.52)	1.85 (2.18-1.63)	10.34 (15.48-7.47)
		10	1.96 (2.33-1.72)	3.24 (3.92-2.77)	2.80 (3.06-2.64)	2.04 (2.43-1.79)	13.27 (21.02-9.34)
90	2.25	5	1.30 (1.57-1.08)	1.11 (1.41-0.84)	2.18 (2.29-2.08)	1.39 (1.69-1.15)	2.22 (3.45-1.44)
		7.5	1.55 (1.89-1.29)	1.44 (1.85-1.12)	2.29 (2.43-2.21)	1.64 (2.02-1.37)	3.45 (5.69-2.21)
		10	1.72 (2.14-1.42)	1.66 (2.16-1.30)	2.38 (2.58-2.28)	1.81 (2.26-1.49)	4.49 (7.48-2.83)

a. For blood perfusion range: nominal (low-high)

The Dimensions are Shown for Nominal Perfusion, and the Parenthetic Range From Zero to High perfusion. The Complete Tabulation Is Found in Table S1

#### 2) Implementation of Fast a Priori Planning

**Fig. 4.**
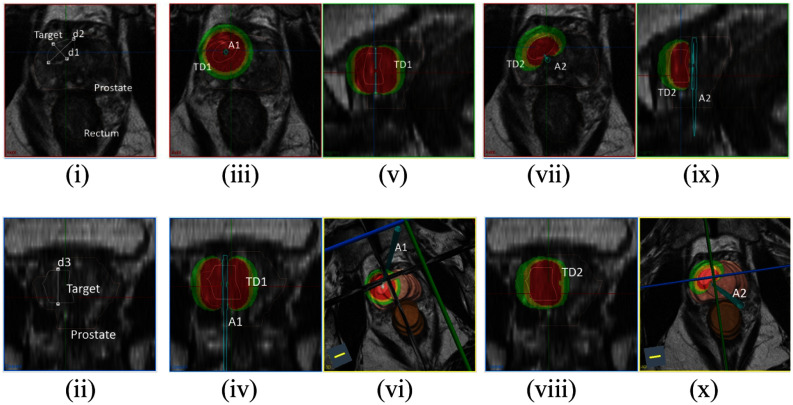
Segmentation of the anatomical volumes and measurement of the target showing in axial (i) and coronal plane (ii). Coverage of intraprostatic lesion using simulated *TD*_43_ = 240 min generated by applicator 360° (iii-vi) and applicator 180° (vii-x) for different insertion and rotation positions, acoustic power and heating time. Lethal thermal dose contours are shown as green 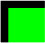, yellow 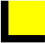, and red 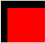 overlays corresponding to simulated perfusion of 0, 2.5, and 5 kg/m^3^/s, respectively. (Axial plane- i, iii, vii, Coronal plane- ii, iv, viii, Sagittal plane- v, ix and 3D view- vi, x).

The 3D lethal thermal dose clouds (*TD*_43_ = 240 mins) and temperature contours after the sonication for each case tabulated in the lookup table are stored as “NRRD objects”. Based on Table S1, applicators data sets can be iteratively selected and positioned by user in implant form within the 3D volume to best achieve maximum coverage of the target(s) and avoidance of sensitive normal tissues during pre-procedure planning or a priori planning. For example, as shown in Fig. 4, after segmentation of the anatomical volumes on the MP-MRI, to ablate defined intraprostatic target (L) based on the measured dimensions (*d1*= 9.9 mm, *d2* = 17.1 mm, *d3* = 16.2 mm) (Fig. 4(i-ii)), different applicators (360°, 180°) at different positions and various rotation angles (1, 2), acoustic power (*I_0_* = 8 W/cm^2^) and heating time (*t_h_* = 5-7.5 mins) were considered and selected to first visualize the precalculated dose overlap with the target. Fig. 4(iii-vi) illustrates the coverage of *TD*_43_ 240 cloud for applicator 360° at position 1 with *I_0_* = 8 W/cm^2^, *t_h_* = 5 mins, whereas Fig. 4(vii-x) shows the applicator 180° at position 2 directing energy inward with *I_0_* = 8 W/cm^2^, *t_h_* = 7.5 mins. The impact on the coagulation dimensions with respect to variations in blood perfusion from low, moderate, and high blood perfusion values (0, 2.5, and 5 kg/m^3^/s) is delineated by green, yellow, and red zones, respectively. This manual and iterative pre-treatment planning tool provides a fast technique to select a single applicator configuration, positioning and rotation angle, as well as optimize the power and time setting based on the maximum and safe coverage of the targeted lesion.

### C. 3D Patient-Specific Forward Treatment Planning

#### 1) Example Clinical Prostate Ablation Cases

**Fig. 5.**
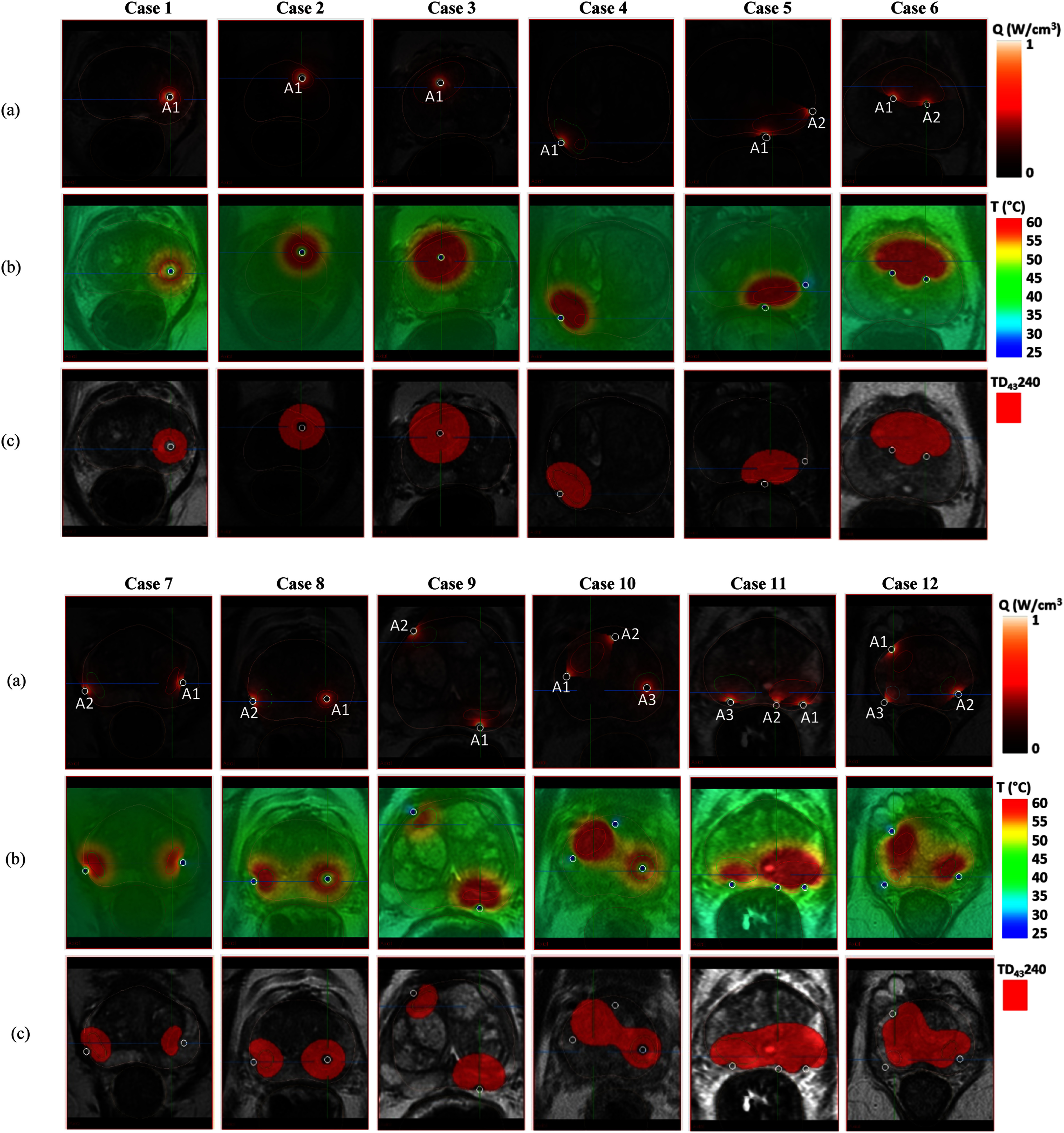
Intraprostatic lesion/s shown for 12 clinical cases and 3D patient-specific thermal treatment models (a) normalized relative power deposition (*Q*, W/cm^3^), (b) temperature (*T*, °C) and (c) lethal thermal dose (*TD*_43_ 240, mins) distribution, shown as a red overlay, in the central transverse heating plane.

A series of clinical cases (n=12), segmented from MP-MRI into models, were used to demonstrate the technology performance and applicability of forward treatment planning for focal, quadrant and hemi-gland prostate ablation using interstitial ultrasound (Fig. 5). For each case the target location and dimensions were considered, and applicator parameters and coverage were selected using Table S1. As demonstrated in the Section III-B (2), using interactive tools for selection, the insertion positioning and applicator directivity angle were adjusted to optimally target defined intraprostatic lesions (L) for conformal thermal ablation and minimize heating to the rectum and urethra (if desired). Two temperature monitoring points, *T_U_* and *T_R_*, were defined near the urethra and rectum respectively. Table IV summarizes each test case and simulated ablation performance with the number of target lesions and respective volumes, selected applicator settings (directionality, transducer elements, intensity, and sonication time), urethral maximum temperature, *T_Umax_* (°C) and rectal maximum temperature, *T_Rmax_* (°C), maximum temperature, *T*_max_ (°C) in the target ROI and ablation volume, *V_abl_* (cm^3^). The resulting power deposition patterns, temperature distributions, and lethal thermal dose overlays are shown on planning MR images representing the central transverse heating plane in Fig. 5 for each test case.

**TABLE IV:** Summary of Trial Configurations and Results for 12 Clinical Cases

Case	Targets	Target Volume ^a^ (cm^3^)	Trial No.	Applicator Setting				Thermal Dosimetry				Thermal Dosimetry w/Urethral Cooling			
				^b^ Configuration	No. of Transducer	Intensity (W/cm^2^)	Sonication time (min)	*T_Umax_* (°C)	*T_Rmax_* (°C)	*T_max_* (°C)	*V_abl_* (cm^3^)	*T_Umax_* (°C)	*T_Rmax_* (°C)	*T_max_* (°C)	*V_abl_* (cm^3^)
1	1	L1: 0.57	#1	L1: A1_360°	1	6	5	43.4	43.4	54.9	1.07	-	-	-	-
2	1	L1: 0.70	#1	L1: A1_360°	2	6	5	50.1	41.6	58.5	4.83	-	-	-	-
			#2	L1: A1_360°	2	6, 4	5	48.6	41.1	57.0	1.99	25.0	39.8	56.7	1.35
3	1	L1: 3.69	#1	L1: A1_360°	2	8	5	55.7	40.3	68.7	10.25	-	-	-	-
			#2	L1: A1_360°	2	8, 6	5	54.0	40.1	66.8	7.70	34.0	39.4	66.6	6.44
4	2	L1: 0.25 L2: 1.13	#1	L1: A1_180°_in L2: A1_180°_in	1	12	5	42.1	44.4	73.9	2.57	-	-	-	-
5	1	L1: 1.01	#1	L1: A1_180°_in L1: A2_90°	1 1	8 8	5 5	44.1	46.7	66.1	2.67	-	-	-	-
6	1	L1: 5.76	#1	L1: A1_180°_out L1: A2_180°_out	2 2	8 8	5 5	40.8	37.5	78.9	10.51	-	-	-	-
7	2	L1: 0.48 L2: 0.48	#1	L1: A1_180°_in L2: A2_180°_in	1 1	8 8	5 5	43.8	43.0	60.3	1.85	-	-	-	-
8	2	L1: 0.54 L2: 0.42	#1	L1: A1_360° L2: A2_180°_in	1 1	6 8	5 5	49.8	46.2	61.1	2.84	43.2	45.7	60.8	2.19
			#2	L1: A1_360° L2: A2_180°_out	1 1	6 8	5 5	45.1	43.7	59.8	2.06	-	-	-	-
9	2	L1: 1.62 L2: 0.87	#1	L1: A1_180°_in L2: A2_180°_in	2 1	8 8	7.5 7.5	45.4	43.4	67.2	6.57	-	-	-	-
10	2	L1: 2.05 L2: 0.89	#1	L1: A1_90° L1: A2_90° L2: A3_360°	2 2 1	8 8 6	5 5 5	52.3	42.1	66.2	7.03	32.9	41.6	65.2	5.65
11	2	L1: 3.28 L2: 2.08	#1	L1: A1_180°_in L1: A2_90° L2: A2_180°_in	2 2 1	8 8 8	5 5 7.5	52.2	47.6	75.6	11.08	40.2	46.1	73.8	8.34
			#2 *	L1: A1_180°_in L2: A2_180°_in	2 1	8 12	7.5 5	43.2 41.9	45.7 43.6	67.3 75.7	5.25 2.78	-	-	-	-
12	3	L1: 0.56 L2: 0.51 L3: 0.37	#1	L1: A1_180°_in L2: A2_180°_in L3: A3_90°_in	1 1 1	8 8 8	5 5 7.5	51.9	45.8	62.4	5.27	31.3	43.2	61.5	3.37
			#2	L1: A1_180°_out L2: A2_180°_out L3: A3_90°_out	1 1 1	8 8 8	5 7.5 10	45.4	44.0	60.2	2.84	-	-	-	-

^a^Target Volume - Target (L): Volume (cm^3^),

^b^Configuration - Target (L): Applicator (A) _Sector (360°/180°/90°)_Directivity (in/out),

*individual heating

As shown in Fig. 5, single target region in case 1 (L1 = 0.57 cm^3^), 2 (L1 = 0.70 cm^3^) and 3 (L1 = 3.69 cm^3^) away from the prostate margin can be ablated by a single 360° applicator centered within the target, and the axial length can be varied as needed by selection of sonicating transducers such as single element for case 1 (*I_0_* = 6 W/cm^2^, *t_h_* = 5 mins) and two element for case 2 (*I_0_* = 6 W/cm^2^, *t_h_* = 5 mins) and case 3 (*I_0_* = 8 W/cm^2^, *t_h_* = 5 mins). Moreover, different powers can be given to the different elements of the same applicator to control the lesion size along the length of the lesion. For example, as shown in Table IV, trial #2 of case 2, acoustic intensities were *I_0_* = 8 W/cm^2^ and *I_0_* = 6 W/cm^2^ for element 1 and 2 respectively which reduces the ablated volume by ∼58% to more tightly conform to the complete target volume (L1). In case 4, two lesions (L1 = 0.25 cm^3^ and L2 = 1.13 cm^3^) were present in right mid medial posterior zone and overlapped to each other. A single element 180° applicator directing inward (*I_0_* = 12 W/cm^2^, *t_h_* = 5 mins) was selected as a suitable option for an additional small focal lesion near to seminal vesicles but not visualized in this plane. Case 5 (L1 = 1.01 cm^3^) and case 6 (L1 = 5.76 cm^3^) contains one lesion site however, due to their shape and size, two directional applicators (180° and 90° in case 5, and two 180°s in case 6) were placed to ablate the target volume while protecting the adjacent anatomical structures, rectum and urethral mucosa respectively. Case 7 (L1 = 0.48 cm^3^ and L2 = 0.48 cm^3^), case 8 (L1 = 0.54 cm^3^ and L2 = 0.42 cm^3^) and case 9 (L1 = 1.62 cm^3^ and L2 = 0.87 cm^3^) were cases with two distinctly separate lesions, which can be effectively ablated by using two applicators individually targeted at small focal lesions (case 7 and 9: two 180° directing inward, case 8: one 180° directing inward and one 360°). For case 10 (L1 = 2.05 cm^3^ and L2 = 0.89 cm^3^), the two targets can be targeted simultaneously (or sequentially) using two 90° applicators (*I_0_* = 8 W/cm^2^, *t_h_* = 5 mins) peripherally implanted to target lesion 1, and a 360° (*I_0_* = 6 W/cm^2^, *t_h_* = 5 mins) centrally placed applicator for lesion 2. For case 11 (L1 = 3.28 cm^3^ and L2 = 2.08 cm^3^), due to multiple lesions the whole posterior peripheral zone was targeted using three applicators, two 180’s and a single 90. Also, another trial (Table IV, case 11: trial #2*) was made with two 180° applicators which were separately used for each lesion and sonicated sequentially to reduce overlap and potential overheating of the rectum. Case 12 was the site of three lesions (L1 = 0.56 cm^3^, L2 = 0.51 cm^3^ and L3 = 0.37 cm^3^) where three applicators (two 180° and one 90°) were used to simultaneously ablate each associated lesion. In all the 12 clinical cases, temperature monitoring points (TMP) were set to measure the maximum temperature near urethra and rectum.

Table IV shows the multiple trials attempted for some cases to illustrate the possibility of different placement and setting of the applicators for improved conformality of thermal ablation and to reduce the heating of the critical surrounding tissue.

#### 2) Example Case - Planning With Urethral Cooling

**Fig. 6.**
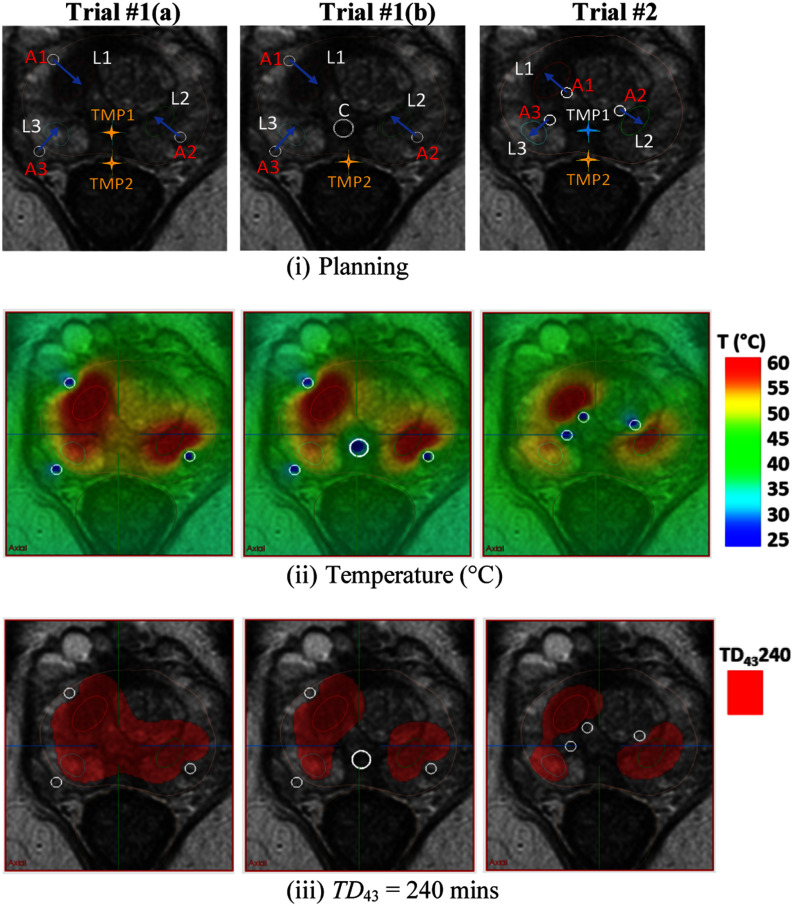
Comparison of ablation configurations including urethral cooling for patient case 12: Peripheral implant for Trial#1(a) A1-A3 directing inward; Peripheral implant for Trial#1(b) with A1-A3 directing inward with integrated urethral cooling; and central implant for Trial #2 with A1-A3 directing outward. Shown are (i) thermal treatment planning based on MP-MRI, (ii) temperature (*T*, °C), and (iii) lethal thermal dose (*TD_43_* 240, mins) distributions, shown as red overlay.

In cases where large volume ablation or targets are close to the urethra, implementing urethral cooling can be used to reduce urethral thermal exposure. As an example, Fig. 6 shows the forward treatment planning for case 12 which required ablation of three distinctly separated intraprostatic lesions. In this case, lesion L1 was located at right mid anterior transition zone (vol. 0.56 cm^3^), lesion L2 was at left mid lateral posterior zone (vol. 0.51 cm^3^) and lesion L3 was at right apical lateral posterior zone (vol. 0.37 cm^3^). Fig. 6 shows the three different planning strategies. Trial #1(a) shows a multi-applicator implant in the periphery of the gland directing energy inward to produce a large volume “hockey stick” ablation of the lesions and adjacent prostate. In trial #1(b), urethral cooling (C) was integrated using the same configuration and operating parameters as (a), and alternatively in trial #2 the applicator positioning was changed to more central and energy was directed outward, with each showing cooling of the central prostate and urethral mucosa. The details of acoustic power and heating time for each trial are listed in Table IV, Case 12. In trial #1(a), the TMP1 temperature at the urethral mucosa reached 51.9 °C. The alternative strategies with urethral cooling (trial #1(b)) or changing the positioning and directivity of the applicators to “outward” (trial #2) reduces the TMP1 to less than 45 °C, i.e., gives lower urethral and rectal temperatures.

#### 3) Planning Temperature-Based Feedback Control

**Fig. 7.**
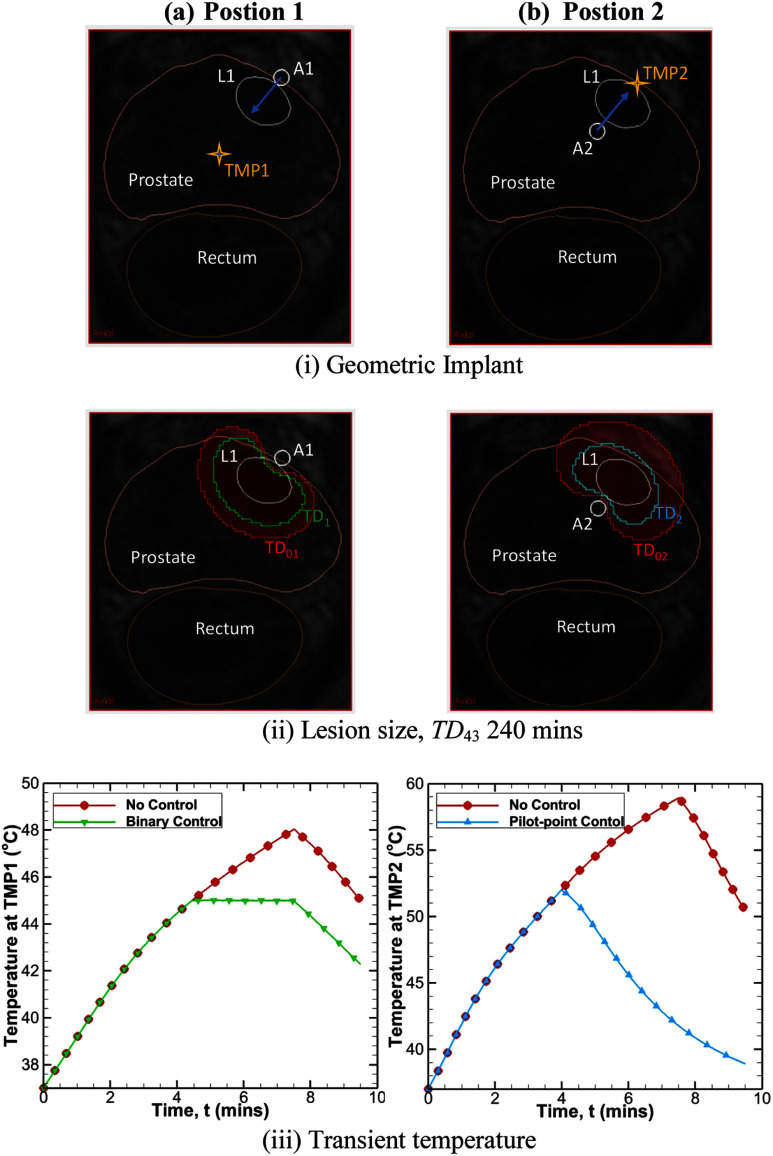
Ablation of lesion L1 where applicator A is placed at (a) position 1 directing energy inward with binary power control at location TMP1, and (b) position 2 directing energy outward with pilot-point power control at location TMP2. Overall duration of applicator applied power under control was 7.5 min and set to zero thereafter. Geometric implant of applicator and TMP on MP-MRI (i), comparison of ablated lesion size (ii) and transient temperature profile (iii) with and without feedback for position 1 and 2. (*TD_01_* (red) and *TD_02_* (red) represents the *TD*_43_ 240 mins without control at position 1 and 2 and *TD_1_* (green) and *TD_2_* (blue) represents the *TD*_43_ 240 mins with control at position 1 and 2 respectively.).

Examples of the potential of using modeling for assessment and guiding implementation of invasive temperature feedback control to improve ablation localization and/or safety is shown in Fig. 7. Case 1 shows the difference of no control (constant power, set time) compared to binary power control using a temperature measurement point (TMP1) within the field, in this case a safety point in tissue placed adjacent to the urethra in close proximity to where the outer boundary of the heating zone is anticipated (Fig. 7(a)). In this peripheral implant configuration, with the energy directed toward the urethra, applicator A1 (*I_0_* = 8 W/cm^2^ and *t_h_* = 7.5 min) operated open loop produces an ablation volume of 4.24 cm^3^ and maximum urethral temperature of 49 °C, whereas implementation of the safety point binary control decreased applied power after 4 min to ∼3 W/cm^2^ (∼62% reduction in power) and subsequently reduced ablation volume to 2.49 cm^3^ while still encompassing the target lesion while keeping the urethral zone ≤45 °C. The maximum temperature in the target for this case without and with control were 63.1 °C and 59.1 °C respectively. Case 2 compares no control to the use of pilot-point control to end treatment or power delivery once a lethal temperature elevation (52 °C) is reached just outside the target boundary, as a means of avoiding unnecessary thermal destruction outside the prostate capsule (Fig. 7(b)). In this central implant configuration, with the energy directed toward the target and outer boundary of the prostate, applicator A2 (*I_0_* = 8 W/cm^2^ and *t_h_* = 7.5 min) operated open loop produces an ablation volume of 5.05 cm^3^ and extends approximately 6 mm outside the capsule, whereas implementation of the pilot point control reduced the overtreatment margin to ∼ 1 mm with a reduced volume of 2.26 cm^3^ which completely covered the target volume. For this case maximum temperature within the target without and with control were 66.6 °C and 61.3 °C respectively. For both cases, the positioning of the applicator and TMP relative to the target lesion and prostate anatomy is shown in Fig. 7(i), lethal thermal dose contours *TD*_43_ = 240 min with no control *TD_0__1_* (red) and *TD_02_* (red) is compared to control *TD_1_* (green) and *TD_2_* (blue), respectively in Fig. 7(ii), and temperature at TMP as a function of treatment time in Fig. 7(iii).

## IV. Discussion

Treatment planning is an important and critical tool for guiding prostate thermal interventions. The present study develops a comprehensive set of treatment planning tools tailored for the implementation of interstitial ultrasound applicators for selective prostate ablation. Applicators with sectored transducers (e.g., 90° and 180°) provide directional energy patterns which can be oriented to produce shaped ablation zones conforming to the target area, while affording protection of adjacent tissues. Moreover, the number of elements within the applicator is independently adjustable, enabling tailoring of the heating length to optimally fit the target. Treatment planning is particularly advantageous in ensuring the protection of critical organs, such as the urethra, rectum, and neurovascular bundle (NVB), from unintended thermal damage or exposure. a priori treatment planning, based upon a lookup table of applicator use parameters to predicted thermal damage zones, along with graphical user interface tools allowing manipulation of 3D renderings of the applicator configuration and damage zones within 3D patient image-based space. This provides a fast method to select the optimal operating parameters and applicator/catheter placement, and mostly applicable to single device implants. Full forward 3D treatment planning can be based upon the actual implant, as explored in clinical patient specific cases, demonstrates applicability of workflow and technique and to develop heating strategies using single or multiple CBUS device implants. The planning is anticipated to be performed by interventional radiologists or urologists, with assistance from a medical physicist or technician. These treatment planning tools leverage the unique spatial control of interstitial ultrasound applicators in conjunction with the 510(k) cleared TheraVision delivery system and streamline pre-selection of applicator configurations and plan positioning to facilitate precision thermal ablation within the prostate gland.

A rapid method of a priori planning was developed by generating a library of 3D thermal dose distributions corresponding to interstitial ultrasound applicator configurations and parameters that bracket the range as intended for clinical use in prostate. This a priori planning can be first applied using pre-procedure MRI/US or initial intra-procedure US imaging exams to determine optimal placement (trajectory angle, direction, insertion depth) to target. The interstitial ultrasound applicator can be inserted percutaneously under US/EM navigation for guidance and real-time position monitoring into or close to the planned positioning and rotation angle. Based upon measurements of the target region from pre-procedure or intra-procedure imaging studies, applicator configurations (number of elements, directivity) and operating parameters (acoustic power output, duration) from Table S1 along with intuitive approximate placement can be used to define the nominal device. Further, the thermal dose clouds provide a 3D visualization tool of the therapy zone within the patient anatomy (Fig. 4), allowing for fine-tuning of the optimal trajectory and rotation angle of the applicator, and confirmation that the configuration and settings are appropriate. In order to accommodate unknown blood perfusion and dynamic variations which lead to uncertainty in the simulations, isodose clouds are interposed for low, moderate, and high perfusion values (0–5 kg/m^3^/s) to visualize the range of predicted damage zones. Clinician decision, based upon experience and/or doppler or MRI blood flow information, can make an assessment of the relative perfusion and use to lookup initial operating parameters and device selection, and then manipulated for positioning to account for target coverage and ensuring safety profiles using real-time intra-procedure TRUS image or pre-treatment using MP-MRI (Fig. 4). This a priori treatment planning technique is applicable to single applicator implants of focal lesions, or multi-applicator implants where each of the applicators are targeting a separate isolated defined intraprostatic lesions (L) with approximately 1–2 cm separation.

Forward treatment planning of interstitial prostate thermal therapy using patient specific models is an important tool for implant design and developing treatment delivery strategies which is practical for complicated implants with diffuse lesions or multi-focal disease and can be applied pre-procedure or intra-procedure (but with the caveat of required simulation time). For the verification study, numerical simulations were performed for the parameters used in experiments given in the references [41], [55] and computed isodose contours were compared to visibly thermal coagulated tissues. The reasonably good match between the experimental and computational results (Table II), establishes the correctness of the present numerical methodology applied to treatment planning. Prostate specific thermal and acoustic parameters based on [43], [60] are incorporated into these models. In this work we employed a constant acoustic attenuation coefficient following the treatment modeling and planning studies by [47], [61] and as verified with *in vivo* [59]. The precise nature of dynamic changes to attenuation and absorption during thermal coagulation are not well defined, but based upon earlier modeling studies that incorporated these changes the maximum temperatures could be higher and the ablation volumes slightly smaller [43]. A series of patient-specific thermal treatment plans representative for interstitial ultrasound ablation of a broad range of T2/T3 prostate disease (Fig. 5), have demonstrated the applicability for applying conformal focal, quadrant, hemi-gland, and “hockey-stick” or “dog-leg” ablation as needed [33]. Table IV exemplifies various cases with either single or multiple CBUS device implants for ablating small focal lesions to larger contiguous volumes from ∼ 1 cm^3^ to 11 cm^3^. Some of the key findings from these planning studies include applicator placement strategies and optimal configurations and control parameters to target lesions of various dimensions and locations within the prostate while limiting exposure to critical surrounding tissue such as the NVB's, urethral mucosa, and rectum. These studies reinforce and augment earlier modeling [41], [42], [43], [46], [47], *in-vivo* [29], [62] and clinical data [31] which demonstrate conformability of the interstitial ultrasound heating patterns to the target region, along with the potential use of directional sectored applicators and positioning of the acoustic dead zone to afford tissue protection. In this setting, treatment planning can determine placement and configurations of peripheral implants with directional applicators adjacent to sensitive tissues (e.g., rectum), with the active sector directed inward to target focal lesions (Fig. 5, Table IV: Case 4, 7, 9, 11). Alternatively, planning can be applied to optimally configure and position applicators medially for 360° heating or shaped directional heating directed toward the peripheral gland or target region, while avoiding normal tissue and sensitive structures (Fig. 5, Table IV: Case 1, 2, 3, 6). Further, this forward treatment planning can be used to estimate the temperature and thermal dose exposure of the urethral mucosa for a given implant configuration, and then to explore various placement and sonication strategies as options to reduce thermal exposure if clinically desired.

In order to ablate large volumes and notably with multiple applicators, the maximum urethral temperature (*T_Umax_*) could be higher than a conservative limit (>45 °C), albeit for short duration and less than lethal thermal dose exposure (Fig. 6, Table IV). Through planning, medial placement of directional applicators aimed peripherally or outward away from the urethra can be developed on a specific case basis to lower urethral mucosa exposure to safer levels (Fig. 6: trial #2). In cases where the target margin is close to the urethra or for large target volumes where inward directed applicators are warranted, a transurethral cooling catheter could be implemented in the plan to afford additional protection (Fig. 6: trial #1(b)).

Apart from standard open-loop operation, interstitial prostate ablation procedures are amenable to placement of invasive temperature probes which can be used to provide temperature feedback for control of acoustic power and treatment duration. In this situation, the patient specific planning provides a useful tool for determining the ideal location of the temperature probe, as well as the efficient threshold temperature and type of control to apply. The location of temperature monitoring points (TMPs), based on positioning of implanted temperature sensors (e.g., needle probes or multijunction thermocouple probes within a 16 g implant catheter [58]), can be defined to model treatment feedback control in the desired patient plan, with a selection of a binary controller for continuous power modulation or a pilot point controller to stop treatment. The binary controller within the planning module mimics an ideally tuned PID controller which is present on the TheraVision delivery platform. In considering the use of binary control (system PID control), the control TMP can be placed in an area of anticipated maximum temperature, for example designated to control therapy power delivery to maintain *T_reg_* = 80 °C or placed at the lesion boundary for example to maintain *T_reg_* = 50 °C for a time sufficient to accrue a lethal thermal dose. During intra-procedure imaging, the values to be used for regulated temperature can be estimated from overlays of a priori isothermal distributions or full treatment planning on the real-time ultrasound, to best accommodate actual sensor positions and to achieve the intended therapy zone. TMPs for pilot point control can be positioned to define the outer boundary of treatment (i.e., 52-54 °C, Fig. 7), or for safety points (i.e., 45 °C, Fig. 7), and to stop the power application and procedure once the threshold is reached as a means to prevent over-treatment. For more complicated implants, a combination of binary control and pilot point control can be used simultaneously.

The developed patient-specific forward treatment planning has the flexibility to allow the design of single or multiple TMPs and thresholds for temperature feedback control and can be integrated with the temperature monitoring and feedback control of the TheraVision delivery system. EM navigation systems, such as incorporated in TheraVision and many other tools for prostate interventions, can be used to place applicators and temperature probes in pre-planned trajectories. This planning tool could be combined with robotic devices [63], similar to those being developed for brachytherapy [64], [65], [66] and ablation [67], [68] for more precise positioning of interstitial applicators within the prostate. In addition, MR thermometry [69], [70] or possibly MST-CBE [71], acoustic radiation force impulse imaging (ARFI), or contrast enhanced ultrasound imaging methods [72] for intra-procedure monitoring can offer the additional advantage of real-time visualization and quantification of temperature changes within the tissue. After completion of the treatment procedure, the user can analyze the thermal coagulation patterns achieved during treatment using post-procedure imaging studies and follow-up to assess the efficacy and completeness of tissue ablation. By comparing the intended treatment plan (computed thermal dose) with the actual thermal outcomes observed, the accuracy of device positioning and directivity, as well as treatment planning, can be evaluated.

Moving forward, enhancements to this treatment planning could include the improvement in computational time with hardware acceleration and also with software efficiency enhancement. Integrating GPU acceleration into the computational engine should significantly enhance the speed of the calculations. Once the computation efficiency and speed are increased, incorporating an optimization based inverse planning method, such as incorporated for interstitial ultrasound hyperthermia [30], [42], RF or MW thermal ablation [73], [74] or brachytherapy implants [75] could be considered to provide optimal positioning, applied power levels, and duration at the time of clinical procedure to meet patient-specific clinical objectives such as maximizing tumor coverage and minimizing damage to surrounding healthy tissues. This integration can enhance the precision and efficacy of interstitial ultrasound thermal ablation procedures while also facilitating fast treatment planning with optimization-based automated selection and placement of applicators. In addition, the treatment planning tools developed in the present study rely mostly on visualization of thermal dose and temperature profiles overlaid on anatomy to best fit and ensure adequate target coverage (plus margin) and with avoidance of sensitive structures. Enhancements to the a priori fast planning, which is based on iterative manipulation of pre-calculated thermal dose clouds, could include real-time display of percent gross tumor target volume, clinical target volume (tumor and margin), and normal tissue volume covered by lethal thermal dose, with safety distinctions for the rectum, urethra, and NVBs. The forward treatment planning could incorporate more detailed quality metrics such as thermal dose-volume and temperature – volume histograms for all target and normal tissue volumes, with percent volume coverage of tumor and clinical target volumes to required thermal dose clearly delineated, as well as below safety thresholds for sensitive normal tissues.

## V. Conclusion

The present study establishes an advancement in treatment planning methodologies for selective prostate ablation utilizing catheter-based ultrasound interstitial applicators. The developed treatment planning tools can be utilized as a guidance in the placement and direction of single or multiple applicators to effectively ablate focal, multifocal and diffuse intraprostatic lesions. Planning for feedback control with the right selection of the temperature monitoring points can be used to control power and hence, improve conformality of thermal ablation and reduce heating in the critical surrounding tissue. Furthermore, this study also demonstrated the applicability of workflow and technique by exploring clinical patient specific-cases and the critical considerations to strategize heating using single or multiple CBUS device implants with temperature-based feedback controls.

*Conflict of Interest Statement:* There is no conflict of interest. ECB and TH are employees of Acoustic MedSystems, Savoy, IL

*Authors Contribution:* Conceptualization: CJD, ECB; Funding acquisition: CJD, ECB; Investigation: PG, TH, CJD, ECB; Methodology: PG, TH, CJD, MZ; Patient data collection: CJD, I-CH; Writing original draft: PG, CJD; Writing-review and editing: PG, CJD, TH, ECB, MZ, I-CH.

## Supplementary Materials

Supplementary materials
