# Supplementary material for: Treatment Planning Strategies for Interstitial Ultrasound Ablation of Prostate Cancer
Source: IEEE Open J Eng Med Biol. 2024 May 8;5:362–75. doi: 10.1109/OJEMB.2024.3397965 (PMC11186654; doi:10.1109/OJEMB.2024.3397965)
Supplement: Supplementary materials [file supp1-3397965.docx]

TABLE S1

lookup table for selection of applicators, listing dimensions of the thermal coagulation zone (*TD*_43_ 240-600 min) for applicator directivity, acoustic power and time over the range of blood perfusion. The dimensions are shown for nominal perfusion, and the parenthetic range from zero to high

| Acoustic Power | No. of Transducer | Heating time (min) | Lesion Dim. (cm)^a^  *TD*_43_ = 600 min | | | Radial Dist. (cm)^a^  *TD*_43_ = 240 min |
| --- | --- | --- | --- | --- | --- | --- |
|  |  |  | *d_1_* (Rad.) | *d_2_* (Width) | *d_3_* (Ax. L) | *R* |
| **Applicator 360** | | | | | | |
| *P_a_* = 6 W  (*I_0_* = 8 W/cm^2^) | 1 | 5 | 1.00(1.17-0.87) | - | 1.06(1.16-0.97) | 1.07(1.25-0.94) |
|  |  | 7.5 | 1.17(1.40-1.00) | - | 1.20(1.37-1.09) | 1.25(1.50-1.08) |
|  |  | 10 | 1.29(1.57-1.09) | - | 1.31(1.56-1.16) | 1.36(1.68-1.16) |
|  | 2 | 5 | 1.19(1.41-1.02) | - | 2.28(2.41-2.21) | 1.27(1.50-1.08) |
|  |  | 7.5 | 1.44(1.74-1.24) | - | 2.47(2.66-2.34) | 1.53(1.84-1.29) |
|  |  | 10 | 1.62(1.97-1.39) | - | 2.62(2.90-2.45) | 1.69(2.08-1.43) |
|  | 3 | 5 | 1.28(1.51-1.09) | - | 3.48(3.59-3.40) | 1.36(1.61-1.16) |
|  |  | 7.5 | 1.57(1.87-1.34) | - | 3.67(3.86-3.55) | 1.64(1.97-1.40) |
|  |  | 10 | 1.77(2.14-1.50) | - | 3.82(4.10-3.65) | 1.84(2.25-1.56) |
|  | 4 | 5 | 1.27(1.51-1.07) | - | 4.68(4.81-4.61) | 1.34(1.61-1.15) |
|  |  | 7.5 | 1.59(1.90-1.34) | - | 4.85(5.07-4.75) | 1.66(1.99-1.40) |
|  |  | 10 | 1.79(2.19-1.53) | - | 5.02(5.30-4.85) | 1.87(2.28-1.59) |
| *P_a_* = 9 W  (*I_0_* = 12 W/cm^2^) | 1 | 5 | 1.36(1.51-1.24) | - | 1.45(1.56-1.34) | 1.44(1.61-1.29) |
|  |  | 7.5 | 1.55(1.78-1.39) | - | 1.66(1.87-1.52) | 1.63(1.88-1.46) |
|  |  | 10 | 1.69(1.96-1.51) | - | 1.82(2.12-1.64) | 1.77(2.08-1.56) |
|  | 2 | 5 | 1.59(1.80-1.43) | - | 2.68(2.83-2.57) | 1.65(1.88-1.48) |
|  |  | 7.5 | 1.86(2.13-1.67) | - | 2.95(3.17-2.79) | 1.94(2.22-1.72) |
|  |  | 10 | 2.05(2.38-1.84) | - | 3.17(3.48-2.97) | 2.11(2.48-1.89) |
|  | 3 | 5 | 1.67(1.89-1.51) | - | 3.86(4.03-3.78) | 1.74(1.96-1.56) |
|  |  | 7.5 | 1.97(2.25-1.77) | - | 4.17(4.38-4.00) | 2.04(2.35-1.83) |
|  |  | 10 | 2.19(2.52-1.96) | - | 4.38(4.68-4.18) | 2.26(2.64-2.03) |
|  | 4 | 5 | 1.67(1.89-1.51) | - | 5.09(5.23-4.98) | 1.74(1.98-1.55) |
|  |  | 7.5 | 2.00(2.28-1.79) | - | 5.36(5.59-5.22) | 2.07(2.37-1.85) |
|  |  | 10 | 2.24(2.58-2.00) | - | 5.57(5.86-5.37) | 2.29(2.69-2.05) |
| **Applicator 180** | | | | | | |
| *P_a_* = 3 W  (*I_0_* = 8 W/cm^2^) | 1 | 5 | 0.89(1.09-0.72) | 1.26(1.59-0.91) | 0.82(0.96-0.64) | 0.98(1.19-0.81) |
|  |  | 7.5 | 1.06(1.31-0.86) | 1.56(2.00-1.19) | 0.96(1.11-0.81) | 1.15(1.42-0.94) |
|  |  | 10 | 1.16(1.47-0.95) | 1.75(2.28-1.36) | 1.05(1.24-0.90) | 1.25(1.60-1.03) |
|  | 2 | 5 | 1.06(1.33-0.81) | 1.46(1.98-0.90) | 2.07(2.20-1.89) | 1.15(1.44-0.89) |
|  |  | 7.5 | 1.32(1.65-1.04) | 1.95(2.58-1.41) | 2.22(2.38-2.16) | 1.40(1.76-1.12) |
|  |  | 10 | 1.48(1.88-1.18) | 2.25(3.00-1.68) | 2.34(2.54-2.18) | 1.57(2.00-1.25) |
|  | 3 | 5 | 1.18(1.44-0.96) | 1.75(2.21-1.33) | 3.27(3.40-3.10) | 1.26(1.54-1.03) |
|  |  | 7.5 | 1.44(1.79-1.18) | 2.23(2.87-1.73) | 3.43(3.59-3.28) | 1.53(1.89-1.25) |
|  |  | 10 | 1.63(2.05-1.32) | 2.57(3.34-2.00) | 3.52(3.76-3.37) | 1.72(2.18-1.39) |
|  | 4 | 5 | 1.13(1.43-0.89) | 1.58(2.14-1.02) | 4.47(4.60-4.30) | 1.22(1.54-0.95) |
|  |  | 7.5 | 1.44(1.81-1.14) | 2.15(2.86-1.58) | 4.62(4.78-4.47) | 1.53(1.92-1.21) |
|  |  | 10 | 1.64(2.09-1.31) | 2.56(3.38-1.89) | 4.72(4.91-4.56) | 1.73(2.22-1.38) |
| *P_a_* = 4.5 W  (*I_0_* = 12 W/cm^2^) | 1 | 5 | 1.31(1.48-1.18) | 2.12(2.44-1.88) | 1.22(1.33-1.15) | 1.38(1.56-1.24) |
|  |  | 7.5 | 1.49(1.73-1.32) | 2.44(2.86-2.13) | 1.38(1.55-1.26) | 1.57(1.83-1.39) |
|  |  | 10 | 1.62(1.92-1.41) | 2.66(3.18-2.29) | 1.49(1.73-1.35) | 1.69(2.02-1.48) |
|  | 2 | 5 | 1.52(1.74-1.35) | 2.44(2.84-2.09) | 2.47(2.58-2.38) | 1.59(1.83-1.39) |
|  |  | 7.5 | 1.78(2.08-1.57) | 2.90(3.47-2.50) | 2.65(2.84-2.52) | 1.85(2.18-1.63) |
|  |  | 10 | 1.96(2.33-1.72) | 3.24(3.92-2.77) | 2.80(3.06-2.64) | 2.04(2.43-1.79) |
|  | 3 | 5 | 1.60(1.84-1.42) | 2.62(3.05-2.29) | 3.66(3.77-3.58) | 1.68(1.93-1.49) |
|  |  | 7.5 | 1.90(2.21-1.68) | 3.15(3.74-2.73) | 3.84(4.04-3.72) | 1.97(2.32-1.74) |
|  |  | 10 | 2.11(2.49-1.85) | 3.51(4.26-3.03) | 4.01(4.26-3.83) | 2.18(2.59-1.91) |
|  | 4 | 5 | 1.59(1.84-1.40) | 2.57(3.03-2.19) | 4.84(4.97-4.78) | 1.67(1.94-1.47) |
|  |  | 7.5 | 1.92(2.24-1.68) | 3.14(3.77-2.69) | 5.05(5.24-4.92) | 1.99(2.34-1.74) |
|  |  | 10 | 2.14(2.54-1.88) | 3.55(4.31-3.01) | 5.20(5.46-5.02) | 2.22(2.65-1.94) |
| *P_a_* = 6 W  (*I_0_* = 16 W/cm^2^) | 1 | 5 | 1.56(1.73-1.44) | 2.63(2.95-2.40) | 1.49(1.62-1.41) | 1.63(1.83-1.50) |
|  |  | 7.5 | 1.77(2.00-1.60) | 2.99(3.42-2.68) | 1.69(1.88-1.57) | 1.84(2.10-1.66) |
|  |  | 10 | 1.90(2.19-1.72) | 3.05(3.77-2.88) | 1.83(2.08-1.68) | 1.98(2.32-1.77) |
|  | 2 | 5 | 1.80(2.01-1.63) | 3.00(3.41-2.69) | 2.73(2.85-2.63) | 1.86(2.11-1.69) |
|  |  | 7.5 | 2.07(2.35-1.88) | 3.49(4.06-3.12) | 2.97(3.17-2.83) | 2.14(2.45-1.93) |
|  |  | 10 | 2.27(2.61-2.04) | 3.86(4.53-3.41) | 3.16(3.45-2.96) | 2.33(2.72-2.09) |
|  | 3 | 5 | 1.88(2.10-1.70) | 3.18(3.60-2.85) | 3.93(4.06-3.83) | 1.95(2.19-1.76) |
|  |  | 7.5 | 2.19(2.49-1.98) | 3.74(4.32-3.33) | 4.18(4.38-4.03) | 2.26(2.59-2.04) |
|  |  | 10 | 2.42(3.07-2.19) | 4.12(5.45-3.68) | 4.35(4.74-4.18) | 2.48(3.17-2.23) |
|  | 4 | 5 | 1.88(2.11-1.70) | 3.14(3.60-2.80) | 5.14(5.27-5.05) | 1.95(2.19-1.76) |
|  |  | 7.5 | 2.21(2.52-2.00) | 3.75(4.35-3.33) | 5.37(5.59-5.24) | 2.28(2.62-2.05) |
|  |  | 10 | 2.46(2.84-2.20) | 4.19(4.90-3.68) | 5.55(5.83-5.36) | 2.53(2.93-2.26) |
| *P_a_* = 7.5 W  (*I_0_* = 20 W/cm^2^) | 1 | 5 | 1.75(1.92-1.63) | 3.03(3.34-2.78) | 1.71(1.84-1.61) | 1.83(2.00-1.69) |
|  |  | 7.5 | 1.97(2.21-1.81) | 3.15(3.84-3.02) | 1.94(2.15-1.81) | 2.05(2.31-1.87) |
|  |  | 10 | 2.13(2.42-1.93) | 3.67(4.21-3.31) | 2.12(2.39-1.93) | 2.21(2.53-1.99) |
|  | 2 | 5 | 2.00(2.20-1.83) | 3.40(3.82-3.10) | 2.96(3.09-2.85) | 2.06(2.29-1.89) |
|  |  | 7.5 | 2.29(2.57-2.09) | 3.95(4.48-3.58) | 3.23(3.45-3.08) | 2.35(2.66-2.14) |
|  |  | 10 | 2.50(2.84-2.29) | 4.32(4.97-3.90) | 3.43(3.73-3.24) | 2.56(2.93-2.33) |
|  | 3 | 5 | 2.08(2.30-1.91) | 3.58(4.01-3.28) | 4.16(4.30-4.05) | 2.15(2.39-1.97) |
|  |  | 7.5 | 2.41(2.71-2.19) | 4.18(4.75-3.78) | 4.43(4.66-4.28) | 2.47(2.79-2.25) |
|  |  | 10 | 2.64(3.00-2.41) | 4.60(5.29-4.16) | 4.65(4.94-4.46) | 2.71(3.10-2.46) |
|  | 4 | 5 | 2.08(2.32-1.92) | 3.57(4.01-3.24) | 5.36(5.52-5.26) | 2.15(2.39-1.97) |
|  |  | 7.5 | 2.44(2.73-2.23) | 4.19(4.79-3.78) | 5.64(5.85-5.48) | 2.50(2.83-2.27) |
|  |  | 10 | 2.69(3.06-2.45) | 4.67(5.36-4.19) | 5.84(6.09-5.65) | 2.76(3.15-2.48) |
| **Applicator 90** | | | | | | |
| *P_a_* = 2.25 W  (*I_0_* = 12 W/cm^2^) | 1 | 5 | 1.15(1.33-1.01) | 1.00(1.19-0.83) | 0.98(1.06-0.89) | 1.24(1.45-1.07) |
|  |  | 7.5 | 1.30(1.56-1.13) | 1.19(1.47-0.99) | 1.08(1.18-0.99) | 1.39(1.68-1.20) |
|  |  | 10 | 1.42(1.74-1.19) | 1.32(1.67-1.09) | 1.14(1.31-1.04) | 1.51(1.84-1.29) |
|  | 2 | 5 | 1.30(1.57-1.08) | 1.11(1.41-0.84) | 2.18(2.29-2.08) | 1.39(1.69-1.15) |
|  |  | 7.5 | 1.55(1.89-1.29) | 1.44(1.85-1.12) | 2.29(2.43-2.21) | 1.64(2.02-1.37) |
|  |  | 10 | 1.72(2.14-1.42) | 1.66(2.16-1.30) | 2.38(2.58-2.28) | 1.81(2.26-1.49) |
|  | 3 | 5 | 1.42(1.68-1.22) | 1.30(1.59-1.06) | 3.38(3.48-3.31) | 1.50(1.79-1.29) |
|  |  | 7.5 | 1.69(2.05-1.43) | 1.65(2.06-1.35) | 3.50(3.64-3.41) | 1.77(2.17-1.49) |
|  |  | 10 | 1.87(2.31-1.57) | 1.88(2.40-1.52) | 3.60(3.78-3.48) | 1.96(2.43-1.65) |
|  | 4 | 5 | 1.38(1.67-1.14) | 1.19(1.54-0.91) | 4.59(4.68-4.50) | 1.46(1.79-1.21) |
|  |  | 7.5 | 1.68(2.05-1.38) | 1.59(2.05-1.24) | 4.69(4.85-4.61) | 1.76(2.18-1.46) |
|  |  | 10 | 1.88(2.35-1.55) | 1.86(2.43-1.47) | 4.79(4.96-4.68) | 1.97(2.48-1.62) |
| *P_a_* = 3 W  (*I_0_* = 16 W/cm^2^) | 1 | 5 | 1.44(1.63-1.30) | 1.41(1.61-1.25) | 1.20(1.30-1.15) | 1.53(1.73-1.38) |
|  |  | 7.5 | 1.63(1.87-1.44) | 1.63(1.91-1.43) | 1.34(1.46-1.24) | 1.71(1.99-1.52) |
|  |  | 10 | 1.74(2.05-1.53) | 1.77(2.12-1.53) | 1.41(1.60-1.30) | 1.83(2.17-1.61) |
|  | 2 | 5 | 1.64(1.89-1.45) | 1.59(1.88-1.38) | 2.43(2.53-2.36) | 1.72(1.98-1.51) |
|  |  | 7.5 | 1.89(2.22-1.67) | 1.94(2.31-1.67) | 2.58(2.73-2.48) | 1.98(2.33-1.73) |
|  |  | 10 | 2.08(2.46-1.81) | 2.17(2.63-1.85) | 2.69(2.92-2.54) | 2.16(2.58-1.88) |
|  | 3 | 5 | 1.74(1.99-1.54) | 1.76(2.03-1.54) | 3.62(3.72-3.55) | 1.82(2.09-1.62) |
|  |  | 7.5 | 2.03(2.36-1.79) | 2.13(2.52-1.85) | 3.77(3.94-3.67) | 2.11(2.48-1.85) |
|  |  | 10 | 2.23(2.64-1.95) | 2.39(2.87-2.05) | 3.88(4.13-3.19) | 2.31(2.76-2.01) |
|  | 4 | 5 | 1.72(2.00-1.51) | 1.69(1.99-1.46) | 4.82(4.92-4.76) | 1.79(2.09-1.57) |
|  |  | 7.5 | 2.04(2.39-1.77) | 2.11(2.53-1.80) | 4.99(5.13-4.87) | 2.11(2.50-1.84) |
|  |  | 10 | 2.25(2.69-1.95) | 2.41(2.92-2.04) | 5.01(5.31-4.96) | 2.33(2.80-2.03) |
| *P_a_* = 3.75 W  (*I_0_* = 20 W/cm^2^) | 1 | 5 | 1.66(1.84-1.53) | 1.69(1.87-1.53) | 1.39(1.51-1.32) | 1.74(1.94-1.58) |
|  |  | 7.5 | 1.85(2.10-1.68) | 1.93(2.21-1.74) | 1.53(1.71-1.44) | 1.94(2.21-1.74) |
|  |  | 10 | 1.98(2.31-1.78) | 2.11(2.44-1.87) | 1.65(1.87-1.52) | 2.07(2.41-1.84) |
|  | 2 | 5 | 1.87(2.11-1.69) | 1.93(2.19-1.72) | 2.62(2.73-2.51) | 1.95(2.21-1.75) |
|  |  | 7.5 | 2.14(2.46-1.93) | 2.29(2.65-2.03) | 2.79(2.97-2.67) | 2.23(2.57-1.99) |
|  |  | 10 | 2.34(2.71-2.08) | 2.54(2.98-2.24) | 2.92(3.18-2.78) | 2.41(2.83-2.14) |
|  | 3 | 5 | 1.97(2.21-1.78) | 2.07(2.34-1.86) | 3.82(3.92-3.74) | 2.04(2.31-1.84) |
|  |  | 7.5 | 2.27(2.60-2.04) | 2.47(2.86-2.20) | 4.01(4.19-3.88) | 2.34(2.71-2.09) |
|  |  | 10 | 2.48(2.88-2.22) | 2.77(3.25-2.44) | 4.14(4.40-3.99) | 2.57(2.99-2.28) |
|  | 4 | 5 | 1.94(2.23-1.76) | 2.01(2.34-1.80) | 5.00(5.13-4.93) | 2.03(2.32-1.82) |
|  |  | 7.5 | 2.28(2.62-2.05) | 2.48(2.88-2.18) | 5.21(5.37-5.09) | 2.36(2.73-2.10) |
|  |  | 10 | 2.52(2.93-2.24) | 2.80(3.30-2.45) | 5.35(5.57-5.18) | 2.59(3.04-2.30) |

^a^ For Blood Perfusion Range: nominal (low-high)

*P_a_* Acoustic Power

*I_0_* Acoustic Intensity at Transducer Surface
